# Infrared Microspectroscopy and Imaging Analysis of Inflammatory and Non-Inflammatory Breast Cancer Cells and Their GAG Secretome

**DOI:** 10.3390/molecules25184300

**Published:** 2020-09-19

**Authors:** Hossam Taha Mohamed, Valérie Untereiner, Gianfelice Cinque, Sherif Abdelaziz Ibrahim, Martin Götte, Nguyet Que Nguyen, Romain Rivet, Ganesh D. Sockalingum, Stéphane Brézillon

**Affiliations:** 1 Laboratoire de Biochimie Médicale et Biologie Moléculaire, Université de Reims Champagne-Ardenne, 51097 Reims, France; hotaha@msa.eun.eg (H.T.M.); romain.rivet@univ-reims.fr (R.R.); 2 CNRS UMR 7369, Matrice Extracellulaire et Dynamique Cellulaire—MEDyC, 51097 Reims, France; 3 Zoology Department, Faculty of Science, Cairo University, Giza 12613, Egypt; isherif@sci.cu.edu.eg; 4 Faculty of Biotechnology, October University for Modern Sciences and Arts, Giza 12613, Egypt; 5 Université de Reims Champagne-Ardenne, PICT, 51097 Reims, France; valerie.untereiner@univ-reims.fr; 6 MIRIAM Beamline B22, Diamond Light Source, Harwell Campus, Chilton-Didcot OX11 0DE, UK; gianfelice.cinque@diamond.ac.uk (G.C.); que.nguyen@diamond.ac.uk (N.Q.N.); 7 Department of Gynecology and Obstetrics, Münster University Hospital, 48149 Münster, Germany; martingotte@uni-muenster.de; 8 Université de Reims Champagne-Ardenne, BioSpecT-EA7506, 51097 Reims, France; ganesh.sockalingum@univ-reims.fr

**Keywords:** inflammatory breast cancer, glycosaminoglycans, proteoglycans, secretome, infrared (micro)spectroscopy, imaging, synchrotron-FTIR

## Abstract

Glycosaminoglycans (GAGs)/proteoglycans (PGs) play a pivotal role in the metastasis of inflammatory breast cancer (IBC). They represent biomarkers and targets in diagnosis and treatment of different cancers including breast cancer. Thus, GAGs/PGs could represent potential prognostic/diagnostic biomarkers for IBC. In the present study, non-IBC MDA-MB-231, MCF7, SKBR3 cells and IBC SUM149 cells, as well as their GAG secretome were analyzed. The latter was measured in toto as dried drops with high-throughput (HT) Fourier Transform InfraRed (FTIR) spectroscopy and imaging. FTIR imaging was also employed to investigate single whole breast cancer cells while synchrotron-FTIR microspectroscopy was used to specifically target their cytoplasms. Data were analyzed by hierarchical cluster analysis and principal components analysis. Results obtained from HT-FTIR analysis of GAG drops showed that the inter-group variability enabled us to delineate between cell types in the GAG absorption range 1350–800 cm^−1^. Similar results were obtained for FTIR imaging of GAG extracts and fixed single whole cells. Synchrotron-FTIR data from cytoplasms allowed discrimination between non-IBC and IBC. Thus, by using GAG specific region, not only different breast cancer cell lines could be differentiated, but also non-IBC from IBC cells. This could be a potential diagnostic spectral marker for IBC detection useful for patient management.

## 1. Introduction

Glycosaminoglycans (GAGs) are unbranched and negatively charged heteropolysaccharides consisting of repeating disaccharide units of alternating uronic acids and N-acetylated hexosamine [1]. The majority of GAGs are covalently attached to core proteins to form proteoglycans (PGs) [2]. PGs are present at the cell surface, in the extracellular matrix (ECM), intracellular granules and basement membranes [3]. PGs are involved in several biological functions, where they modulate cell growth-factor activation, regulate collagen fibrillogenesis, affect tumor cell growth and invasion, and influence corneal transparency [3]. GAGs and PGs represent one of the major macromolecules of the ECM [1] and play important roles in cancer progression, where changes in their expression and enzymes involved in their biosynthesis and/or degradation occur [1]. For instance, chondroitin sulfate proteoglycans (CSPGs) were shown to activate the extracellular signal-regulated kinase and focal adhesion kinase in melanoma [4]. Decorin, a major dermatan sulfate proteoglycan (DSPG), was demonstrated to regulate epidermal growth factor receptor (EGFR) signaling, thus controlling proliferation in melanoma [5]. Syndecans, acting in harmony with integrins and hyaluronan signaling through CD44, were shown to increase cancer cell motility [6]. Moreover, Syndecan-1 regulates the adhesion of cancer cells to lymphatic vessel endothelium [7]. Degradation of cell-surface heparan sulfate (HS) chains and matrix heparan sulfate proteoglycans (HSPGs) by heparanase increases invasion and metastasis [8]. Inhibition of perlecan expression decreases the growth of colon carcinoma cells and tumor angiogenesis [9]. CS has an antiangiogenic effect by inhibiting the migration of transendothelial monocytes [5]. In breast cancer, overexpression of hyaluronan synthase 2 increases ErbB2-dependent signaling leading to disease progression [10], while its suppression leads to an inhibition of tumorigenesis and progression in breast cancer [11]. High expression level of Syndecan-1 in breast cancer patients correlated with poor prognosis, an aggressive phenotype and weak response to neoadjuvant chemotherapy [12]. In addition, overexpression of Glypican-1 has been found to be associated with high-grade breast cancer tissues [13].

Inflammatory breast cancer (IBC) is the most lethal form of breast cancer with a higher incidence in young women [14]. IBC is known to exhibit poor prognosis and a low survival rate in comparison with other breast cancer types [15]. Clinically, IBC is characterized by the presence of positive metastatic lymph nodes and up to 30% of IBC patients exhibit metastasis at diagnosis [16]. Pathologically, IBC is identified by the presence of dermal and stromal tumor emboli. The incidence of lymphatic tumor emboli prevents drainage of the lymph fluid leading to swelling of the breast tissue and causing the inflammatory nature of the IBC [17]. Previously, Syndecan-1 was shown to modulate the cancer stem cell phenotype via the IL-6/STAT3, Notch and EGFR signaling pathways in triple-negative IBC [18]. CSPGs and their biosynthetic pathways play a major role in aggressive breast cancer metastasis, thus considering promising targets for antimetastatic-therapies [19]. PGs play essential physiological and pathological roles during cellular development, proliferation, differentiation, and cancer metastasis [20,21]. The GAG chains are able to interact with molecules such as growth factors which are essential for cell differentiation and maintenance of tissue organization during embryo development but also during tumor progression. Analysis of side-chain composition of GAGs isolated from malignant breast tissues indicates an elevation in CS [19,22,23,24]. An increase in CS-A and CS-E sulfation sequences and a decrease in CS-C and CS-D have been previously described in breast cancer [25,26,27]. Thus, the sulfate groups present on CS play a pivotal role in the cellular processes involved in the progression of breast cancer [1,24,28]. CS sulfation patterns had differing effects for different breast cancer cell types, and the greatest inhibitory effect was observed for the most aggressive, triple negative breast cancer cell line MDA-MB-231 [24]. Similarly, differences in the sulfation of HS were recently shown to have an impact on signal transduction, proliferation and invasion of breast cancer cells [29].

Due to the lack of IBC specific biological markers, the characterization of GAGs at cellular and tissue levels in IBC becomes important to understand the diverse biological roles of GAGs in disease progression. Thus, non-invasive screening methods need to be developed to evaluate GAGs as potential diagnostic biomarkers and/or as therapeutic targets in several human cancers, including IBC.

Vibrational spectroscopy can be such a tool. It is an analytical approach used in the diagnosis of many diseases including cancer as it has the ability to detect subtle biochemical changes prior to any morphological changes, which translates into a modification of the spectral profile [30]. These modifications are related to alterations in the concentration and the conformation of functional groups associated with cell components such as nucleic acids, proteins, lipids, carbohydrates or macromolecules present in the extracellular matrix such as collagen, elastin, PGs, small leucine-rich proteoglycans (SLRPs), and GAGs. Indeed, vibrational microspectroscopies have shown promises in characterizing and discriminating types of GAGs despite their close molecular structures and some characteristic spectral regions and peaks were identified [31,32,33].

The potential of vibrational spectroscopy for cell studies has been demonstrated for breast cancer cells. Recently, Fourier Transform InfraRed (FTIR) spectroscopy was used to identify biochemical changes during the progression of breast cancer bone metastasis [34]. Smolina and Goormaghtigh demonstrated that both gene expression level and FTIR spectroscopy are multivariate techniques that give sufficient information to discriminate between thirteen breast cancer cell lines grown in 2D or 3D laminin-rich extracellular matrix (lrECM) cultures [35]. HT-FTIR spectroscopy determined the metabolic modifications induced in MDA-MB-231 cells by numerous polyphenols using unsupervised and supervised statistical analysis [36]. FTIR microspectroscopy could also differentiate between benign, atypical hyperplasia and malignant breast cells with high accuracy. IBC is characterized by a late diagnosis but with the advantages of IR as an ‘optical biopsy’ technique, this can help histopathologists in their decision in IBC diagnosis and decrease the delay between diagnosis and treatment [37].

Recently, our group has shown that vibrational spectroscopy can differentiate non-IBC and IBC at cell and tissue levels [30].

Moreover, spectral signatures of GAGs were identified in cells expressing different levels of GAGs, both at the population and single-cell levels. The obtained spectral data were analyzed by hierarchical cluster analysis (HCA) to characterize these different cell types exhibiting from low to high levels of GAG synthesis [31]. GAG spectral signatures could also be identified by Raman microspectroscopy in live cells and from their conditioned media (CM) [38,39]. Thus, vibrational spectroscopy methodologies were demonstrated to be a useful approach for screening and identifying cells that exhibit a different capacity for GAG synthesis. They are rapid, non-destructive, non-contact and label-free methods. They are able to give a complete “molecular fingerprint” of the studied sample as they are highly sensitive to the structure, composition, and environment of the molecules constituting the studied specimen.

In this study, we employed a combination of FTIR spectroscopic or imaging modalities, including synchrotron-FTIR microspectroscopy, to investigate non-IBC human breast cancer cell lines of different molecular subtype (MDA-MB-231—basal; MCF7—luminal; and SKBR3—Her2) and IBC cells (SUM149), exhibiting varying capacities of GAG synthesis, as well as GAG extracts from their CM, defined here as their secretome.

Compared to our previous studies, this investigation focuses on the GAG absorption region to evaluate the capacity of vibrational spectroscopy modalities to discriminate between IBC and non-IBC via their secretome as well as at the single-cell level.

Exploratory chemometrics was employed for unsupervised data analysis, and a specific spectral signature was identified as a possible biomarker, that could differentiate cell types with different GAG content in their secretome, and delineate IBC from non-IBC cells, thus making it an interesting diagnostic marker that could be useful for breast cancer patient management.

## 2. Results

### 2.1. Quantification of Sulfated GAGs in Breast Cancer Cells Conditioned Media

In order to assess the total amount of sulfated GAGs synthesized by each breast cancer cell type, a Blyscan^TM^ assay was performed on the respective extracted GAGs. Table 1 shows the concentration of sulfated GAGs secreted in the CM of the four breast cancer cell lines, respectively.

The Blyscan^TM^ assay results showed that MCF7, MDA-MB-231, SKBR3 and SUM149 cells were capable of synthesizing and secreting detectable levels of sulfated GAGs in their CM (0.70 ± 0.03 µg/mL, 0.77 ± 0.01 µg/mL, 0.37 ± 0.01 µg/mL, and 0.44 ± 0.03 µg/mL, respectively). It can be noticed that SUM149 IBC cells secreted similar levels of sulfated GAGs as compared to SKBR3 cells but nearly two-fold less than MCF7 and MDA-MB-231 cell lines.

### 2.2. High-Throughput FTIR Spectroscopy and Characterization of GAGs Extracted from Cell Conditioned Media

**Figure 1:**
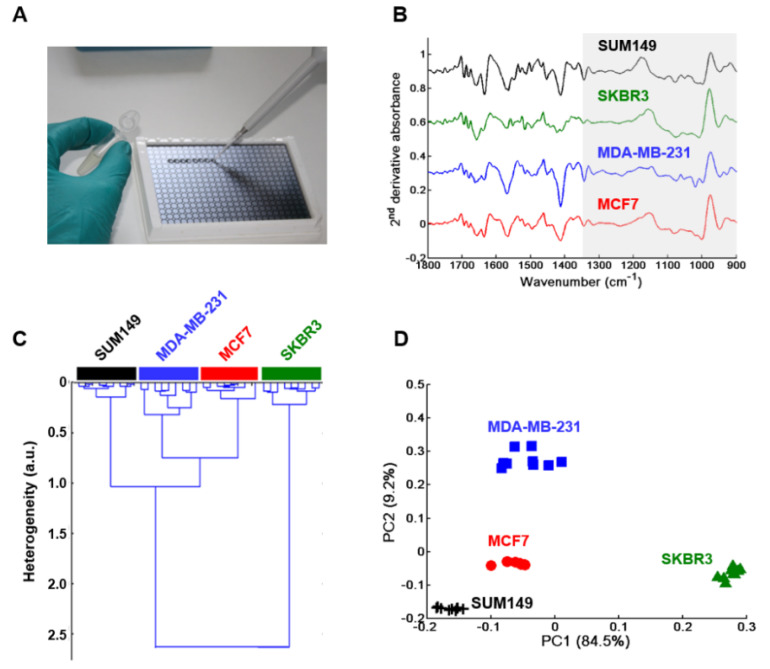
High-throughput Fourier Transform InfraRed (FTIR) spectroscopy of GAGs extracted from conditioned media (secretome) of MCF7, MDA-MB-231, SKBR3 and SUM149 cells. (**A**) Photograph of a silicon plate with sample deposits; (**B**) Comparison between normalized mean second derivative spectra of conditioned media (CM) from the four cell lines. Spectra are offset for clarity; (**C**) HCA analysis and (**D**) PCA score plot of MCF7 (full red circles), MDA-MB-231 (full blue squares), SKBR3 (full green triangles), and SUM149 (black crosses). Both analyses were performed on normalized mean second derivative spectra using the frequency range 1350–900 cm^−1^.

The Blyscan^TM^ assay revealed a difference in the synthesis of sulfated GAGs between the four breast cancer cell lines. In order to have the spectral signatures of GAG extracts obtained from the CM (secretome) of each breast cancer cell type, dried drops were analyzed in toto by high-throughput (HT) FTIR spectroscopy using an IR-transparent silicon multi-well plate (Figure 1A). The advantage of the IR method, compared to the Blyscan^TM^ assay which gives only sulfated-GAGs, is that the vibrational spectrum contains signatures of all secreted GAGs present in the secretome, both sulfated and non-sulfated. The vector normalized second derivative spectra showed some spectral differences between the different breast cancer cell types in the 1800–900 cm^−1^ spectral range (Figure 1B). The HCA cluster analysis performed on the 1350–900 cm^−1^ region corresponding to the GAG absorption range (grey zone in Figure 1B), revealed four distinct groups of spectra corresponding to the four breast cancer cell types (Figure 1C). The dendrogram showed a low intra-group heterogeneity and high inter-group heterogeneity. The data were also analyzed by principal component analysis (PCA). The score plot using the first two PCs (totaling 93.7% of explained variance) (Figure 1D) correctly delineated the four cell lines: MCF7 (red full circles), MDA-MB-231 (blue full squares), SKBR3 (green full triangles) and SUM149 (black crosses). PC2 scores allowed to separate MCF7 from MDA-MB-231 and SUM149 but not from SKBR3 while PC1 scores delineated the latter from MCF7 and from the two other cell lines. This score plot also indicated a low intra-group heterogeneity and high inter-group heterogeneity, allowing a good separation of the extracted GAGs. This corroborates well with the HCA results obtained before.

### 2.3. FTIR Imaging and Characterization of GAGs Extracted from Cell Conditioned Media

**Figure 2:**
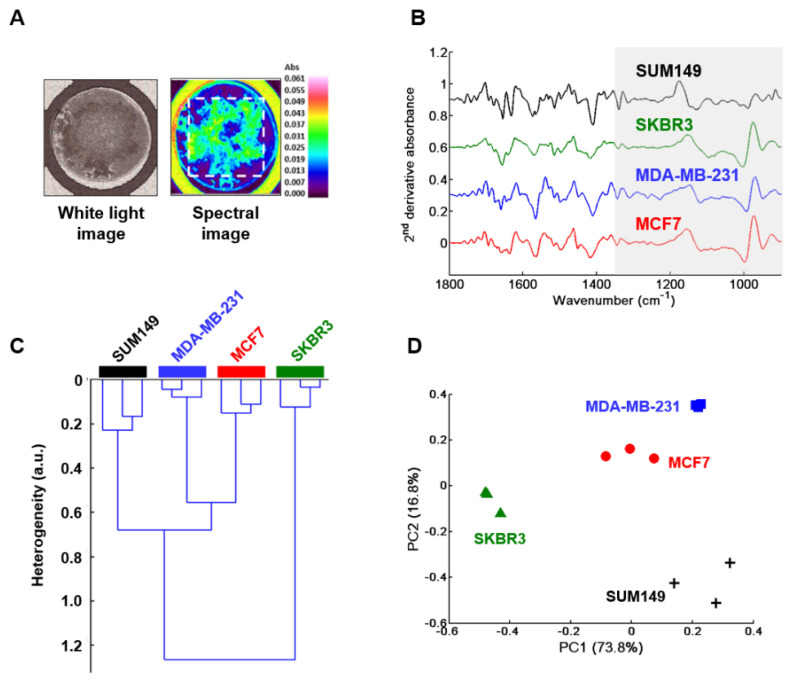
FTIR imaging of GAGs extracted from conditioned media of MCF7, MDA-MB-231, SKBR3 and SUM149 cells. (**A**) Illustration of the white light image of a GAG dried drop (left) and its corresponding spectral image (right); (**B**) Normalized second derivative of the mean spectrum (n = 3) from each cell type. Spectra are offset for clarity; (**C**) HCA analysis and (**D**) PCA score plot of MCF7 (full red circles), MDA-MB-231 (full blue squares), SKBR3 (full green triangles) and SUM149 (black crosses). Both analyses were performed on normalized mean second derivative spectra using the frequency range 1350–900 cm^−1^.

A second approach, based on whole drop FTIR imaging, was performed on three dried spots of GAG extracts obtained from the CM of three independent cell cultures of each cell line (Figure 2A). Mean spectra were processed and they showed similar profiles and a low variability between the different spectra of GAG extracts. Therefore, as for HT-FTIR, normalized mean second derivative spectra were computed to enhance spectral differences.

Figure 2B displays normalized mean second derivative spectra of GAGs extracted from the CM of the four cell lines in the 1800–900 cm^−1^ spectral range, where the GAG absorption region is highlighted in grey. Normalized mean second derivative FTIR spectra were then analyzed by HCA and PCA in the GAGs absorption spectral range 1350–900 cm^−1^. The dendrogram obtained from HCA analysis is, again, in line with the interpretation of low intra-group and high inter-group variabilities, thus enabling differentiation between the GAGs synthesized and secreted by the four breast cancer cell lines (Figure 2C). The PCA score plot using the first two PCs representing 90.6% of total explained variance, is displayed in Figure 2D. The plot confirms low intra-group and high inter-group heterogeneities, and resulted in good discrimination between the GAG extracts obtained from the CM of the four cell types. HT-FTIR spectroscopy and FTIR imaging gave similar results and both allowed good discrimination between the GAGs synthesized and secreted by the four breast cancer cell lines. The good reproducibility between the two experiments on the secretome is illustrated by comparing normalized mean second derivative spectra in Figure S1.

**Figure 3:**
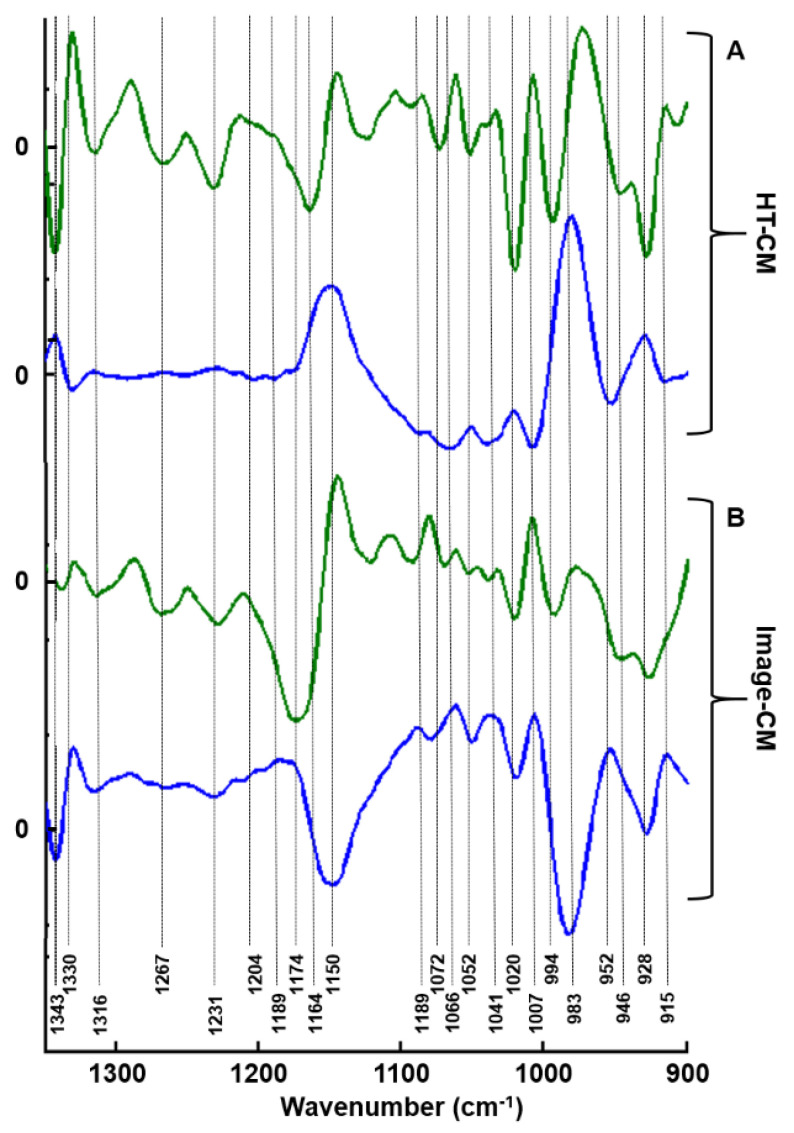
Comparison of first two principal components corresponding to: (**A**) high-throughput FTIR and (**B**) imaging spectra of GAGs extracted from CM. PC1 (blue line) and PC2 (green line).

We further compared the loadings of PC1 and PC2 from both secretome analyses by HT-FTIR and imaging of dried drops (Figure 3).

The two approaches gave very similar information for both PC2 and PC1, with the latter being just a mirror image. This comparison identifies specific bands corresponding to different GAG functional groups [32,33].

### 2.4. FTIR Imaging and Characterization of Single Fixed Breast Cancer Cells

**Figure 4:**
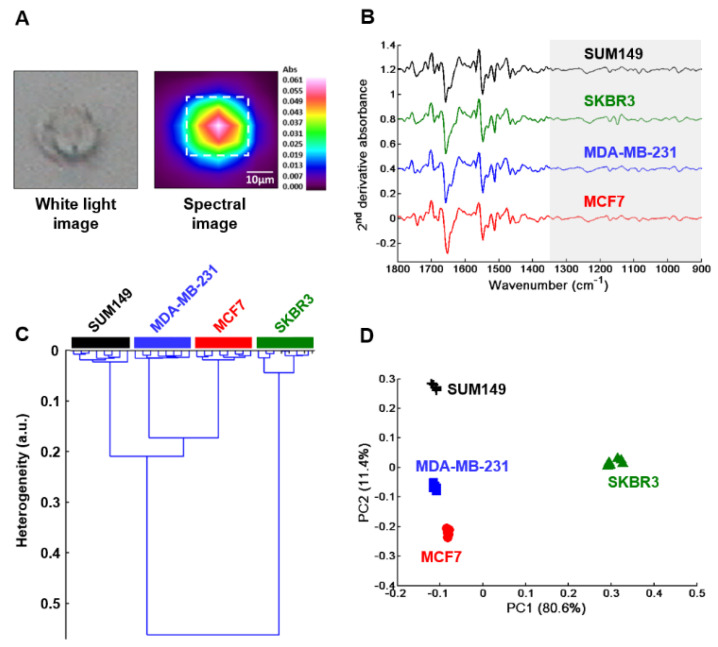
FTIR imaging of MCF7, MDA-MB-231, SKBR3 and SUM149 fixed single cells. (**A**) Illustration of a white light image of MCF7 single fixed cell (left) and its corresponding FTIR image (right). Scale bar: 10 µm; (**B**) Normalized second derivative of mean spectrum (n = 10) of each cell type. Spectra are offset for clarity; (**C**) HCA analysis and (**D**) PCA score plot of MCF7 (full red circles), MDA-MB-231 (full blue squares), SKBR3 (full green triangles) and SUM149 (black crosses). Both analyses were performed on normalized mean second derivative spectra using frequency range 1350–900 cm^−1^.

FTIR imaging was also performed on single fixed whole cells of each breast cancer cell type. An example is shown for the MCF7 cell line in Figure 4A and the three other cell lines are illustrated in Figure S2.

The mean spectrum of each cell was computed from the dotted square (Figure 4A and Figure S2). In a similar way as for GAG extracts, normalized mean second derivative spectra were calculated in 1800–900 cm^−1^ range as shown in Figure 4B. They displayed some modifications between the four cell types in this spectral range. However, for HCA and PCA analyses only the region 1350–900 cm^−1^ (highlighted in grey in Figure 4B) was used to focus on GAGs spectral information. HCA results revealed four well distinct groups corresponding to each cell type and exhibited low intra-group and high inter-group heterogeneities (Figure 4C). The PCA using the first two PCs, representing 92% of total explained variance, is displayed in Figure 4D. The score plot showed low intra-group and high inter-group variabilities resulting in good discrimination of the four cell types corroborating well with the HCA results above.

### 2.5. Synchrotron-FTIR Microspectroscopy and Characterization of Single Fixed Breast Cancer Cells

**Figure 5:**
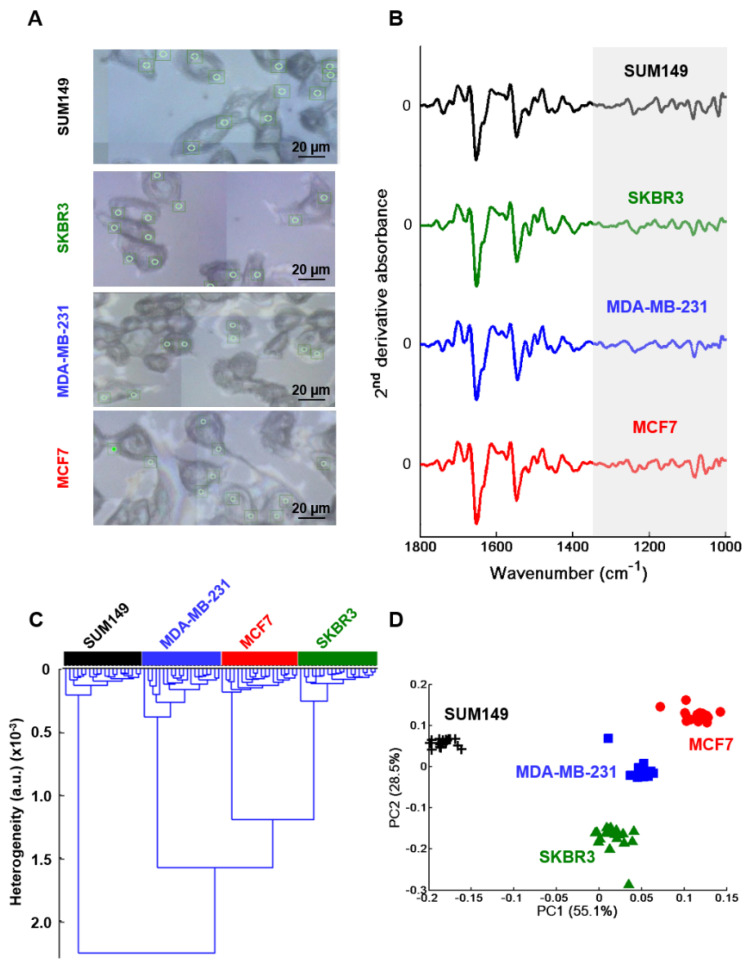
Synchrotron FTIR microspectroscopy of the cytoplasm of MCF7, MDA-MB-231, SKBR3 and SUM149 fixed single cells. (**A**) The white light image of each cell type showing the measurement points (green circle); (**B**) Normalized second derivative mean spectrum (n = 20) from the cytoplasm of the four cell lines. Spectra are offset for clarity; (**C**) HCA analysis and (**D**) PCA score plot of MCF7 (full red circles), MDA-MB-231 (full blue squares), SKBR3 (full green triangles) and SUM149 (black crosses). Both analyses were performed on normalized mean second derivative spectra using frequency range 1350–990 cm^−1^.

The high brightness of the synchrotron IR radiation was sought for to access intra-cellular biochemical information. The measurements were conducted by targeting the cytoplasms of the four cell lines. The rationale for this relies on the fact that GAGs are synthesized in the endoplasmic reticulum, matured in the Golgi apparatus. From there, they can be transported to the cytosol, to the cytoplasmic membrane or secreted in the extracellular matrix. Serglycin remains cytoplasmic and is highly expressed in MDA-MB-231 and moderately expressed in MCF7 breast cancer cells. Therefore, focusing specifically on the cytoplasms by synchrotron-FTIR microspectroscopy appears to be a plausible approach to target cellular spectral markers that can allow differentiation between non-IBC and IBC cell lines, thus helping in finding new markers useful in IBC diagnosis. The Diamond Light Source IR facilities at the MIRIAM B22 beamline was used to analyze the cytoplasms of the four breast cancer cell lines. For each cell line, twenty single cells were selected, and 2 to 3 spectra/cell were collected in their cytoplasms by detecting via slits a spot size of 8 microns. An example of the measured points is illustrated in Figure 5A for each cell line.

Figure 5B displays the mean second derivative spectra corresponding to the four cell lines in the 1800–990 cm^−1^ region. As previously, HCA and PCA analyses were performed on the second derivative spectra in the 1350–990 cm^−1^ spectral range, corresponding to the GAG absorption region. HCA results are displayed in Figure 5C where a grouping of the cell types into specific clusters could be observed. Similar results were obtained by PCA analysis and by plotting the scores of principal components 1 and 2, totaling 83.6% of the explained variance (Figure 5D). The HCA and PCA data not only allowed discrimination between the four breast cancer cell types but also enabled a clear delineation between non-IBC (MCF7, MDA-MB-231 and SKBR3) and IBC (SUM149) cells.

**Figure 6:**
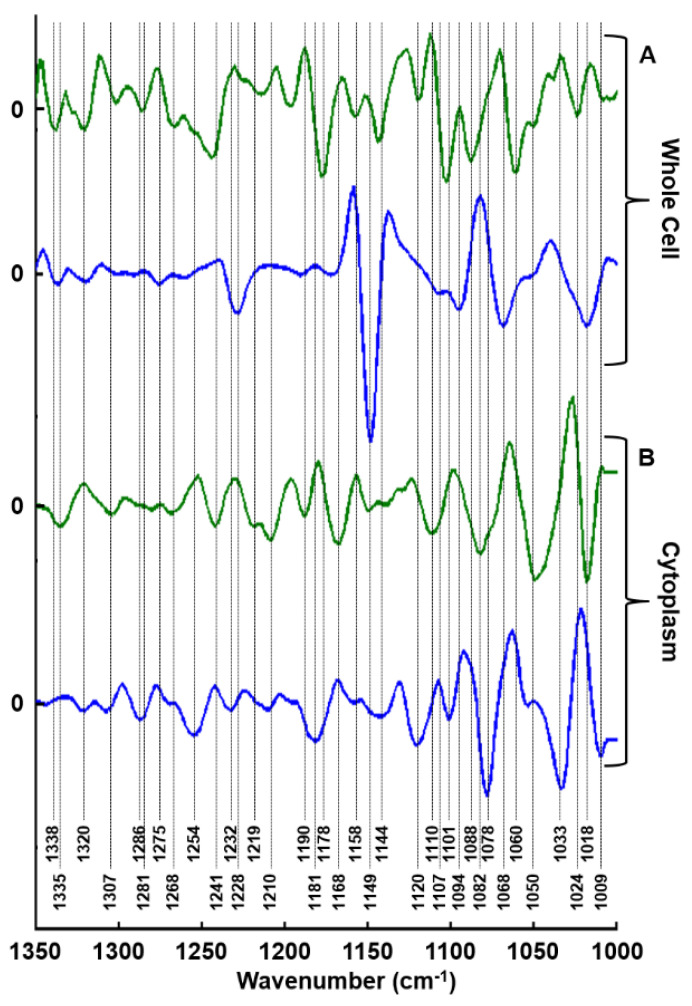
Comparison of first two principal components corresponding to: (**A**) whole cell imaging and (**B**) synchrotron-FTIR microspectroscopy of the cytoplasm. PC1 (blue line) and PC2 (green line).

As for the GAG extracts above, we have compared the PC1 and PC2 loadings from the PCA analysis of the whole cells and cytoplasms (Figure 6).

It can be noticed that the loading vector 1 from cytoplasm measurements exhibits a profile that is different from those observed from extracted GAGs, or loading vector 1 from whole-cell measurements. Some spectral information observed at 1033 cm^−1^, 1078 cm^−1^, 1120 cm^−1^, and 1158 cm^−1^, could be associated with GAG/PGs present in the cytoplasm. Other carbohydrate absorption peaks could also be carried by loading vector 2, as well as cell information.

For each experiment, the mean of second derivative spectra and the standard deviation (SD) were calculated for CM measured by HT-FTIR and FTIR imaging and for single cells measured by FTIR imaging and synchrotron-FTIR. The means are represented by a full line and the SDs by a shaded zone of the same color. It can be noted that all presented data are highly reproducible due to the weak SD. The 16 graphics are shown as supplementary data (Figures S3–S6).

## 3. Discussion

Vibrational microspectroscopy/imaging is an analytical tool used in the diagnosis of many diseases including cancer. It has the ability to detect subtle biochemical changes before any morphological changes, which translate into a modification of the spectral profile [39]. These modifications are related to alterations in the concentration and the conformation of functional groups associated with cell components such as nucleic acids, proteins, lipids, carbohydrates or macromolecules present in the extracellular matrix such as collagen, elastin, PGs, SLRPs, and GAGs. These molecules can be potential biomarkers of specific diseases. Overexpression of CS has been identified in various cancer phenotypes such as prostate, testicular, gastric, pancreatic and breast cancer [28,40,41,42,43,44,45].

Recently, Mohamed and collaborators used vibrational microspectroscopy with chemometric tools such as HCA analysis to discriminate between non-IBC and IBC at cell and tissue levels [30]. The differences in the structure of GAGs/PGs and enzymes involved in their biosynthesis or degradation, modulate cancer progression. Thus, GAGs/PGs represent key players in cancer diagnosis and treatment [1]. Hence, the detection of such biomolecules or any changes in GAGs/PGs structures by vibrational spectroscopy can be helpful in cancer diagnosis, specifically in cancers with no clear biological markers such as IBC [30].

Our study reports on the potential of FTIR spectroscopy and imaging to differentiate cell types based on their capacity to synthesize GAGs and to delineate IBC from non-IBC cells. In the first instance, data reported here on the secretome, clearly indicate reproducible results obtained on dried drops with two different FTIR modalities, HT and imaging, as shown by both HCA and PCA analyses. Indeed, the comparison between the first two loading vectors (PC1 and PC2) of both modalities supports our observations since similar spectral information was obtained. This comparison identifies specific bands corresponding to different GAG functional groups. For instance, the spectral range 1100–980 cm^−1^ contains the C–O–C, C–C–O and C–C–C vibrations of disaccharides and sulfates (OSO_3_^−^). In the range of 1072–1040 cm^−1^, the C–O–C and symmetric (OSO_3_^−^) modes are observed while the asymmetric (OSO_3_^−^) vibrations are present in the 1268–1230 cm^−1^ spectral range [32,33]. In both cases, PC2 loading vector seems to endow the spectral information allowing the differentiation between the secretome of the four cell lines while PC1 delineates SKBR3 secretome from the others (see Figure 3). Since the analysis is based on a specific GAG absorption region, it can be hypothesized that the differences originate from the change in the GAG composition of the secretome and not on cellular components. The secretome contains extracellular vesicles in which chondroitin sulfate proteoglycan 4 (CSPG4) has been detected [46,47]. Notably, SKBR3 belongs to the Her2 subtype, and it has been shown that HS3ST2A-mediated changes in 3-O-sulfation of HS GAGs have a prognostic impact particularly in this subtype of breast cancer [48].

Biochemical assay of sulfated GAGs reveals that MCF7 and MDA-MB-231 content was higher than SKBR3 and SUM149 cells. Blyscan^TM^ is a quantitative method used to measure the sulfation degree of the GAGs in the secretome of the four different cell lines. Results of the biochemical assay of sulfated GAGs revealed that two groups of cells were distinguished based on sulfated GAGs content: one group at high concentration (MCF7 and MDA-MB-231) and one group at lower concentration (SUM149 and SKBR3). Compared to this biochemical assay, vibrational spectroscopy performs better since the four cell lines as well as their secretome were individually well identified. This is probably due to the fact that all GAG information is captured and not only the sulfated ones. FTIR imaging of whole cells led to similar observations as with the secretome concerning PC1 and PC2 (see Figure 3). PC2 carries more spectral information on cell lines and allows them to be separated. In contrast, SKBR3 discrimination based on PC1 could be associated with a difference in GAG composition synthesized by this cell line. In the three previous analyses (GAG extracts dried drops and cell imaging), PC1 allows the separation of the SKBR3 cell line from the others, while PC2 seems to act on cell types differentiation. According to the literature, low PG expression such as Syndecan-1 and CD44 has been detected in SKBR3 comparatively to SUM149 and might explain why it is well delineated from the three other cell lines [18]. This separation from the other cell lines might also be due to other properties like proliferation rather than the invasion capacity of MDA-MB-231 or the inflammatory phenotype of SUM149. These two cell lines, in contrast to MCF7, were described to express CSPG4/NG2, which also contributes to invasiveness [49].

When the cytoplasms are targeted, the results differ slightly as PC1 separates SUM149 (highly inflammatory cells) from the rest and PC2 delineates between cell types. The signal detected in the cytoplasm may be attributed to different GAGs and particularly to GAGs linked to serglycin, an intracellular CSPG. Serglycin is an intracellular PG markedly synthesized by cancer and stromal cells in malignant tissues [50]. Indeed, it is expressed and constitutively secreted by numerous malignant cells, especially in the highly-invasive, triple-negative MDA-MB-231 breast carcinoma cells and is closely linked to a pro-inflammatory gene signature including the chemokine IL-8 [51]. Thus, the discrimination of the four cell lines according to their aggressive mesenchymal phenotype and inflammatory response via synchrotron-FTIR microspectroscopy of cytoplasms might be associated to intracellular serglycin expression. Further works are needed to confirm biochemically the expression of the CSPG serglycin in the four cell lines since its expression in SKBR3 and SUM149 cell lines is not described in the literature. The signal detected in the cytoplasm might also be attributed to GAGs linked to other PGs during their intracellular synthesis and traffic [52]. For example, Listik and collaborators have shown that DS is a GAG that is produced through the epimerization of the glucuronic acid in CS into iduronic acid (IduA) by DS epimerase (DS-epi) 1 and 2 [20]. They have described the expression of DS-epi1 in MCF7, MDA-MB-231, and SKBR3 cell lines, its involvement in cancer progression and showed that the localization of the enzyme in intracytoplasmic organelles may play a decisive role in the tumor growth [20].

By using the GAGs absorption range as a spectral marker, the HCA and PCA analyses of spectral data from HT-FTIR and imaging analysis of the secretome and FTIR imaging of whole single fixed cells, allowed to obtain discrimination between the different cell types. Synchrotron-FTIR microspectroscopy of cytoplasms permitted distinguishing non-IBC from IBC cells. However, the spatial resolution of the beam is not high enough to precisely determine whether the impact of the light was in the endoplasmic reticulum or the Golgi apparatus. To achieve this subcellular resolution, nano-IR spectroscopy could be an interesting alternative to further address the issue of the precise GAGs location within the cytoplasm and the associated epimerase activity. Despite the interesting information was obtained by this approach, there are some limitations that need to be underlined. Firstly, measurements have been performed on fixed cells, while more valuable data from live cells would give a more precise picture of the intracellular metabolism of GAGs synthesis. Secondly, as stated before, intracellular biosynthesis and maturation of GAGs occur in organelles such as the endoplasmic reticulum or the Golgi apparatus. Targeting these subcellular compartments is not achievable even with the high spatial resolution of the synchrotron. Thirdly, although FTIR spectroscopy permits us to have a spectral signature of all GAGs present in the secretome, it cannot directly quantify sulfated GAGs as the Blyscan^TM^ biochemical assay.

Future work will address some of these limitations. For example, FTIR imaging of live cells could be undertaken with specific devices as it has been recently reported [53] but obtaining a good signal to noise in/ex vivo remains a real challenge. Another alternative to live-cell imaging would be to use Raman microspectroscopy [38,39]. In order to overcome the spatial resolution limitation and to reach subcellular organelles, nano-IR spectroscopy (photothermal or AFM-IR) [54] could be an interesting alternative to further address the issue of the precise GAGs location within these organelles and the associated epimerase activity. In order to verify the hypothesis that the spectral signature measured by synchrotron-FTIR microspectroscopy is associated with serglycin expression in the cytoplasm, it would be interesting to confirm this assumption by analyzing serglycin overexpressing cells and their counterparts such as serglycin knockdown cells by biochemical and spectroscopic approaches.

## 4. Materials and Methods

**Figure 7:**
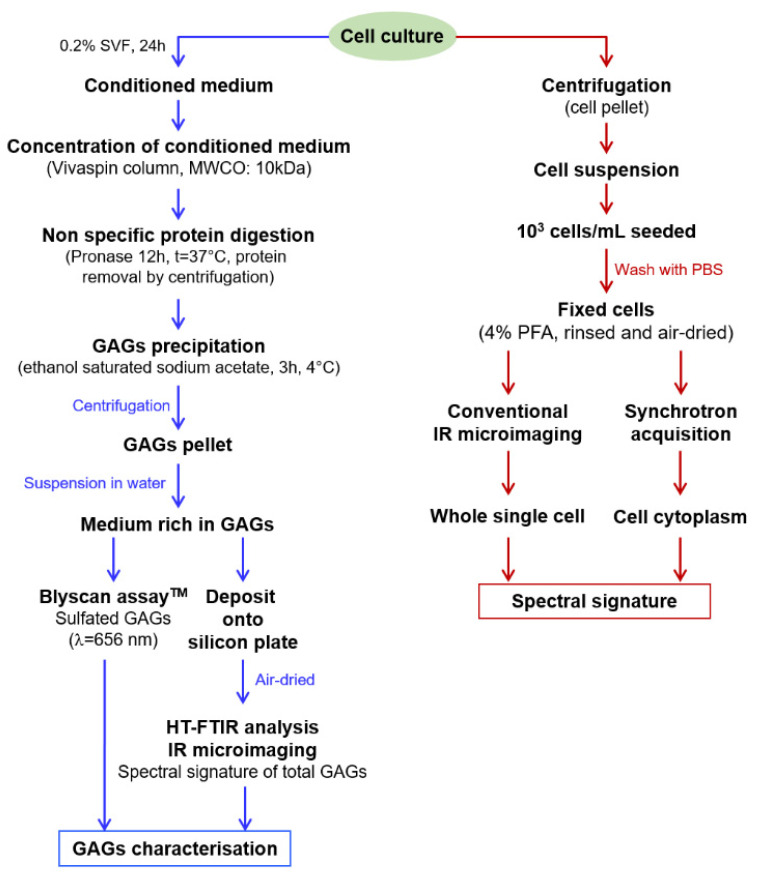
The workflow of the preparation of cells and their conditioned media for biochemical and spectral analyses.

A workflow of the methodological approaches for sample preparation, biochemical assay, HT-FTIR, FTIR imaging and synchrotron-FTIR microspectroscopy, used in this study is illustrated in Figure 7.

### 4.1. Cell Lines

Three non-IBC human breast cancer cell lines MDA-MB-231 (ATCC^®^ HTB-26™), MCF7 (ATCC^®^ HTB-22™), SKBR3 (ATCC^®^ HTB-30™) from LGC Promochem (Wesel, Germany), and one IBC cell line SUM149 (BIOIVT, West Sussex, UK) were used in this study. All cell lines were cultured in DMEM medium with 10% fetal bovine serum and 1% of penicillin/streptomycin antibiotic mixture, except SUM149 cells that were cultured in HAM’s F12 medium with 5% fetal bovine serum, 5 mM HEPES, 1 µg/mL hydrocortisone, 5 µg/mL insulin and 1% of penicillin/streptomycin antibiotic mixture. All cell lines were incubated at 37 °C in 5% CO_2_. Cells were detached at 80% of the confluence with 0.5% trypsin/EDTA (Invitrogen, Illkirch, France). Cells in suspension were centrifuged at 420 *g* for 3 min, then pellets were resuspended. Cell viability was assessed by trypan blue exclusion assay.

### 4.2. Extraction of Glycosaminoglycans from Cells Conditioned Media

Cells CM (10 mL) were collected after 24 h starvation (growth media without FBS). Cell debris in the collected CM were precipitated by centrifugation at 1200× *g* for 4 min. Then, the collected purified CM was concentrated in Vivaspin^TM^ protein concentrator column (Sartorius, Gottingen, Germany) with a cut-off at 10 kDa. Proteins present in the concentrated CM (300 µL) were randomly digested overnight at 37 °C with 0.1 U of pronase (Sigma Aldrich, Saint-Quentin-Fallavier, France). Pronase deactivation was done with the addition of 40 µL NaCl (0.5 M) and incubation at 100 °C for 1 min. After cooling, centrifugation was done for 5 min at 8000× *g* to precipitate the digested proteins. GAGs were precipitated from purified CM by addition of 1350 µL ethanol saturated with sodium acetate and incubated at 4 °C for 3 h. Purified GAGs were precipitated at 8000× *g* for 5 min and air-dried. Dried GAGs were suspended in 40 µL sterile distilled water. The GAG solutions were then studied by both biochemical and infrared analyses. All data obtained were from three independent cultures.

### 4.3. Sulfated Glycosaminoglycans Quantification

Sulfated glycosaminoglycan content was evaluated using the Blyscan™ assay (Biocolor Ltd., Westbury, NY, USA) according to the manufacturer’s instructions. The Blyscan™ dye reagent was added to precipitate the sulfated GAG-dye complex. A sulfated GAG standard (chondroitin 4-sulfate purified from bovine trachea at 100 µg/mL provided in the kit) and the blank reagent (0 µg/mL) were used to produce a calibration curve at 10, 20, 30, 40 and 50 µg/mL. Each GAG extract was prepared by adding 12 µL of sample to 88 µL of 50 mM Tris-HCl buffer pH 7.5, and 100 µL of each dilution were used in the reaction. Five hundred microliters of Blyscan™ dye reagent were added to all tubes and the samples were mixed every 5 min for 30 min at room temperature. The sulfated GAG-dye complex formed was precipitated out from the soluble unbound dye and centrifuged (10 min, 420 *g* at room temperature) to obtain a pellet. The supernatant was discarded and 500 µL of dissociation agent were added. After strong shaking, sulfated GAGs were dissociated from the dye reagent. Then, 200 µL of each sample were withdrawn and loaded in duplicate on a 96-well microplate and ODs were measured at 656 nm on a microplate reader. The concentrations of the sulfated GAGs were then calculated and expressed in micrograms per milliliter of CM.

### 4.4. High-Throughput Infrared Analysis of GAGs Extracted from Conditioned Media

Five µL of GAGs extracted from CM was deposited in triplicate onto a 384 well silicon plate and left to air-dry. The dried plate was analyzed with a Tensor 27 spectrometer coupled to a high-throughput screening HTS-XT extension (Bruker Optics GmbH, Ettlingen, Germany). FTIR acquisitions of the samples were performed in transmission mode, in the spectral range 4000–400 cm^−1^, at a spectral resolution of 4 cm^−1^ with 64 accumulations per spot. Before each sample measurement, the silicon plate background spectrum was recorded and automatically removed from the sample signal. The obtained spectrum is representative of the whole dried spot from each well. Acquisition and pre-processing were performed with the OPUS software (Version 6.0, Bruker Optics, Germany).

### 4.5. FTIR Imaging of Dried Drops of GAG Extracted from Conditioned Media

The re-suspended precipitated GAGs (5 µL) from each cell line was deposited on a multi-well silicon plate and left to air dry as above. FTIR images were acquired in transmission mode using the FTIR microimaging system Spotlight 400 (Perkin Elmer, Courtaboeuf, France) at a spatial resolution of 25 µm/pixel. The spectral range 4000–800 cm^−1^ was used at a spectral resolution of 4 cm^−1^ with 16 accumulations. Spectral images (n = 3) of GAG samples were taken over the whole dried drop, and all spectra were averaged after removing the background spectral contribution from the silicon substrate. The raw spectra were then subjected to an atmospheric correction algorithm to compensate for water vapor and CO_2_ contributions using Spectrum-Image, version 1.6 (Perkin Elmer, Courtaboeuf, France).

### 4.6. FTIR Imaging of Single Fixed Cells

For infrared imaging, the different cell types were plated on a calcium fluoride substrate at 1.5 × 10^4^ cells/mL and allowed to adhere. After 24 to 48 h of cell culture, CaF_2_ substrates were removed from the culture medium and washed three times with Dulbecco’s phosphate buffer saline (DPBS). Cell fixation was performed using 4% paraformaldehyde (PFA) for 30 min at room temperature. Cells were then rinsed with DPBS and distilled water to remove PFA, then air-dried.

Cells (n = 10 cells per cell line) were analyzed by FTIR imaging using the Spotlight 400 system (Perkin Elmer, Courtaboeuf, France). After visual imaging and selection of cells of interest, FTIR imaging was acquired in transmission mode at a spatial resolution of 6.25 µm/pixel, in the spectral range 4000–800 cm^−1^ and a spectral resolution of 4 cm^−1^. Each pixel spectrum corresponded to 128 co-additions.

### 4.7. Synchrotron-FTIR Microspectroscopy of Single Fixed Breast Cancer Cells

In order to specifically target the cytoplasms of the breast cancer cells, synchrotron-based FTIR microspectroscopy was sought and beam time was requested at the Diamond Light Source (DLS) facilities. Breast cancer cells were prepared in the same way as for the above experiments, grown on CaF_2_ windows and fixed with 4% PFA for safe transportation to the Diamond Infrared beamline (MIRIAM B22). There, cell lines were analyzed with a Hyperion 3000 IR Microscope, coupled to a Vertex 80v FTIR Spectrometer, and equipped with a nitrogen-cooled MCT high sensitivity detector (Bruker Optics GmbH). In this configuration, the conventional IR Globar^™^ source was replaced by the synchrotron IR beam, for providing a spectrally broadband and spatially diffraction-limited source. The microscope slits were set to reduce the detected area at the sample to 8 × 8 µm^2^. Samples were visualized in both reflection and transmission modes and cells of interest were identified, located spatially, marked and their cytoplasms measured in the IR transmission mode using a 36× Cassegraian objective. The condenser used was identically at the same magnification. For each cell line, twenty cells were analyzed. Prior to cell spectrum acquisition (n = 3 per cell for each cell line), a background spectrum was recorded in a blank area of the CaF_2_ window in the 4000–800 cm^−1^ spectral range, using a spectral resolution of 4 cm^−1^ and 256 co-additions of the acquisition (circa 30 s per point). Before measuring the background, the condenser focalization was adjusted using the Piezo condenser stage (ANKA-SmartACT, Oldenburg, Germany). All cytoplasm spectra were recorded in the same conditions as the background spectrum. Then, raw spectra were subjected to atmospheric correction to compensate for water vapor and CO_2_ contributions using OPUS 6.5 software (Bruker Optics GmbH).

### 4.8. Preprocessing of Cells and Extracted GAGs Spectral Data and Images

HT spectral data obtained from CM were baseline corrected (elastic method, 9 points), their second derivative spectra computed to increase spectral differences and vector normalized for comparing spectra to each other. For images, mean spectra were calculated from the whole single cells or whole dried drops of GAGs extracted from CM. These were baseline corrected and their second derivative computed and vector normalized. All data were processed with exploratory techniques like HCA (OPUS 6.5 software, Bruker Optics GmbH) and PCA (MATLAB software, MathWorks, Natick, MA, USA). HCA is based on an Euclidean distance calculation and results are represented by a tree diagram called a dendrogram. For PCA, a plot of the scores of the first two principal components, representing a maximum of the total % variance, was used for representing the spectral clusters formed.

## 5. Conclusions

In this study, we have applied HT-FTIR spectroscopy, FTIR imaging and synchrotron-FTIR microspectroscopy to investigate single breast cancer cells from the non-IBC MCF7, MDA-MB-231, SKBR3 and IBC SUM149 cell lines and their corresponding extracted GAGs. The aim was to investigate the potential to differentiate cell types and also between non-IBC and IBC cells using spectral information in the GAGs absorption range. Secretome and whole-cell FTIR microanalysis gave similar results suggesting a cell classification mainly based on GAG content. Synchrotron-FTIR microanalysis of the cytoplasms allowed us to discriminate between non-IBC and IBC. Results indicate a grouping of cell lines possibly on the basis of spectral information from cell--specific intracellular GAG modification during synthesis and trafficking. These findings are interesting as the GAG absorption region can be a promising spectral marker range for the early diagnosis of IBC patients.

## Figures and Tables

**Table 1:** Blyscan™ assay of total sulfated Glycosaminoglycans (GAGs) in conditioned media.

Cell Line	Mean ± SD (µg/mL)
MCF7	0.70 ± 0.03
MDA-MB-231	0.77 ± 0.01
SKBR3	0.37 ± 0.01
SUM149	0.44 ± 0.03

## Supplementary Materials

The following are available online: Figure S1: Comparison between normalized mean second derivative spectra of GAGs extracted from conditioned media of four cell lines obtained by two methods. High-throughput FTIR spectroscopy (full line) and FTIR imaging (dotted line) correspond to the spectra of Figure 1B and Figure 2B, respectively. Spectra are offset for clarity. MCF7 (red curves), MDA-MB-231 (blue curves), SKBR3 (green curves), and SUM149 (black curves).Figure S2: Illustration showing white light images of MDA-MB-231, SKBR3 and SUM149 single fixed cell (left) and their corresponding FTIR images (right). Scale bar: 10 µm. Figure S3: Normalized mean second derivative spectra ± SD of CM-HT-FTIR from (A) MCF7, (B) MDA-MB-231, (C) SKBR3 and (D) SUM149. Figure S4: Normalized mean second derivative spectra ± SD of CM-imaging-FTIR from (A) MCF7, (B) MDA-MB-231, (C) SKBR3 and (D) SUM149. Figure S5: Normalized mean second derivative spectra ± SD of Cell-imaging-FTIR from (A) MCF7, (B) MDA-MB-231, (C) SKBR3 and (D) SUM149. Figure S6: Normalized mean second derivative spectra ± SD of Cell-Synchrotron-FTIR from (A) MCF7, (B) MDA-MB-231, (C) SKBR3 and (D) SUM149.
