# Supplementary material for: Infrared Microspectroscopy and Imaging Analysis of Inflammatory and Non-Inflammatory Breast Cancer Cells and Their GAG Secretome
Source: Molecules. 2020 Sep 19;25(18):4300. doi: 10.3390/molecules25184300 (PMC7570935; doi:10.3390/molecules25184300)
Supplement: Supplementary file 1 [file molecules-25-04300-s001.zip › molecules-893103.pdf]

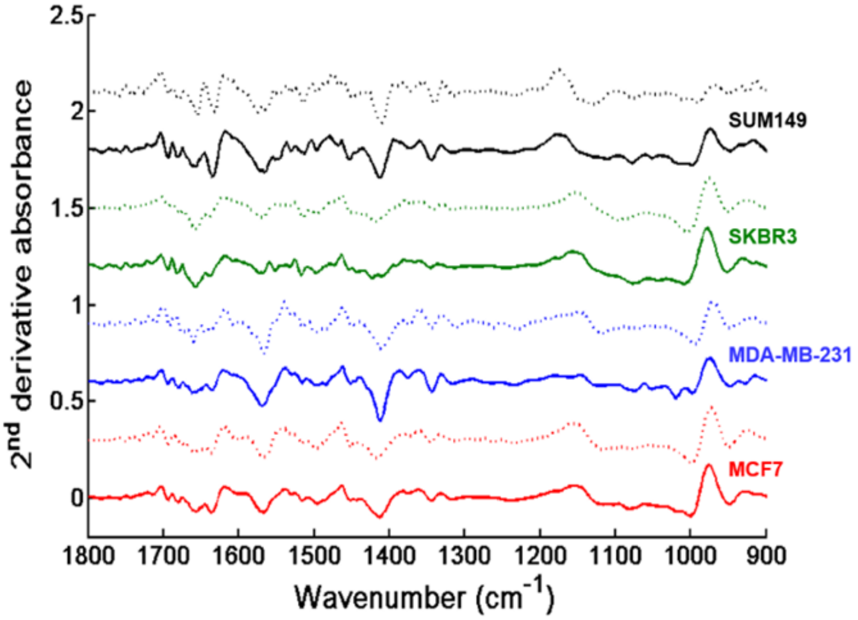

Supplementary Figure 1. Comparison between second derivative spectra of GAGs extracted from conditioned media of four cell lines obtained by two methods. High-throughput FTIR spectroscopy (full line) and FTIR imaging (dotted line) correspond to spectra of Figure 1B and Figure 2B, respectively. Spectra are offset for clarity. MCF7 (red curves), MDA-MB-231 (blue curves), SKBR3 (green curves), and SUM149 (black curves).

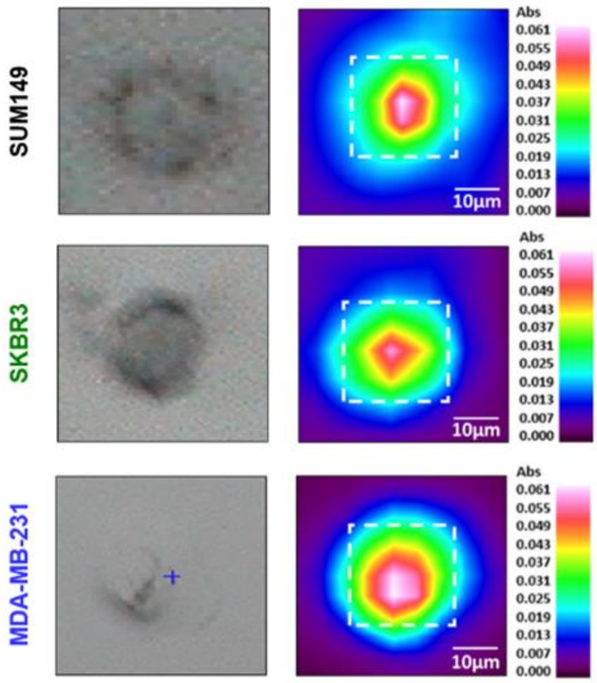

Supplementary Figure 2. Illustration of white light image of MDA-MB-231, SKBR3 and SUM149 single fixed cell (left) and their corresponding FTIR images (right). Scale bar: 10 µm.
