# Supplementary figures and images for: Infrared Microspectroscopy and Imaging Analysis of Inflammatory and Non-Inflammatory Breast Cancer Cells and Their GAG Secretome
Source: Molecules. 2020 Sep 19;25(18):4300. doi: 10.3390/molecules25184300 (PMC7570935; doi:10.3390/molecules25184300)

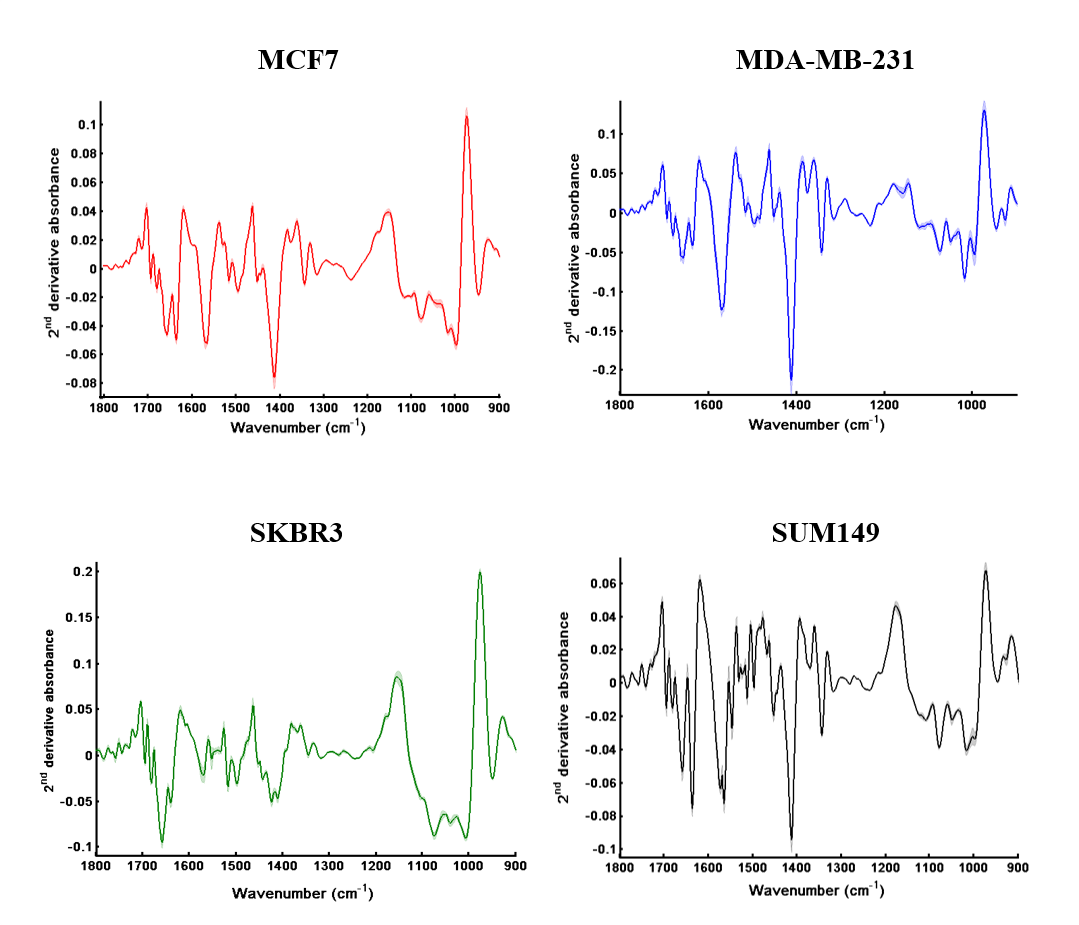

Supplement: Supplementary file 1 [file molecules-25-04300-s001.zip › Figure S3.tif]

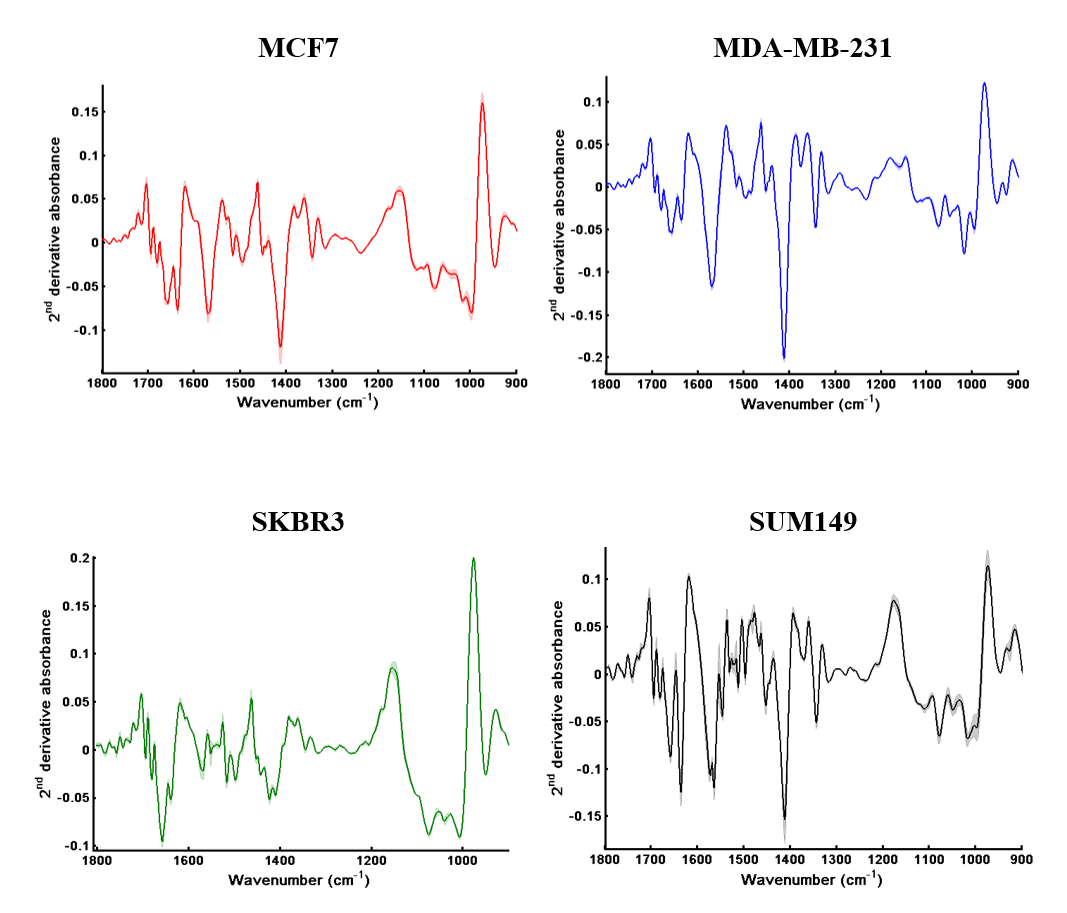

Supplement: Supplementary file 1 [file molecules-25-04300-s001.zip › Figure S4.tif]

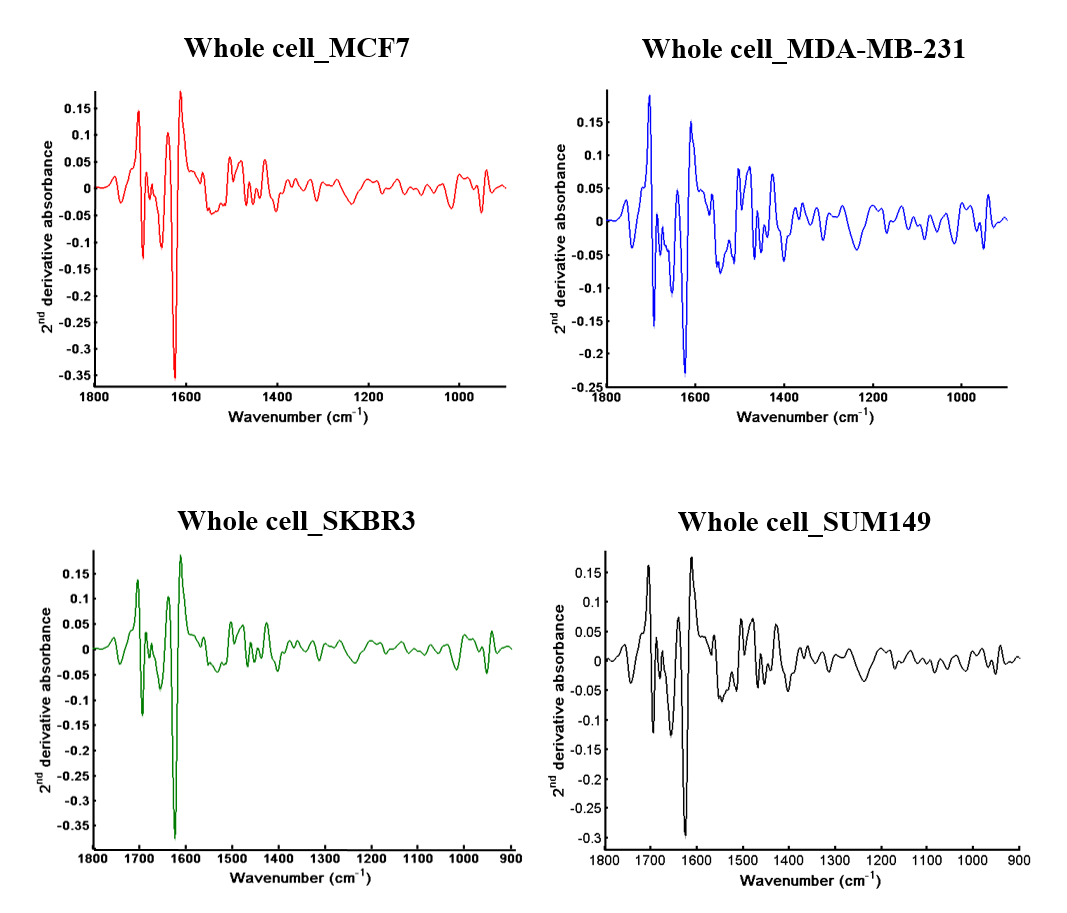

Supplement: Supplementary file 1 [file molecules-25-04300-s001.zip › Figure S5.tif]

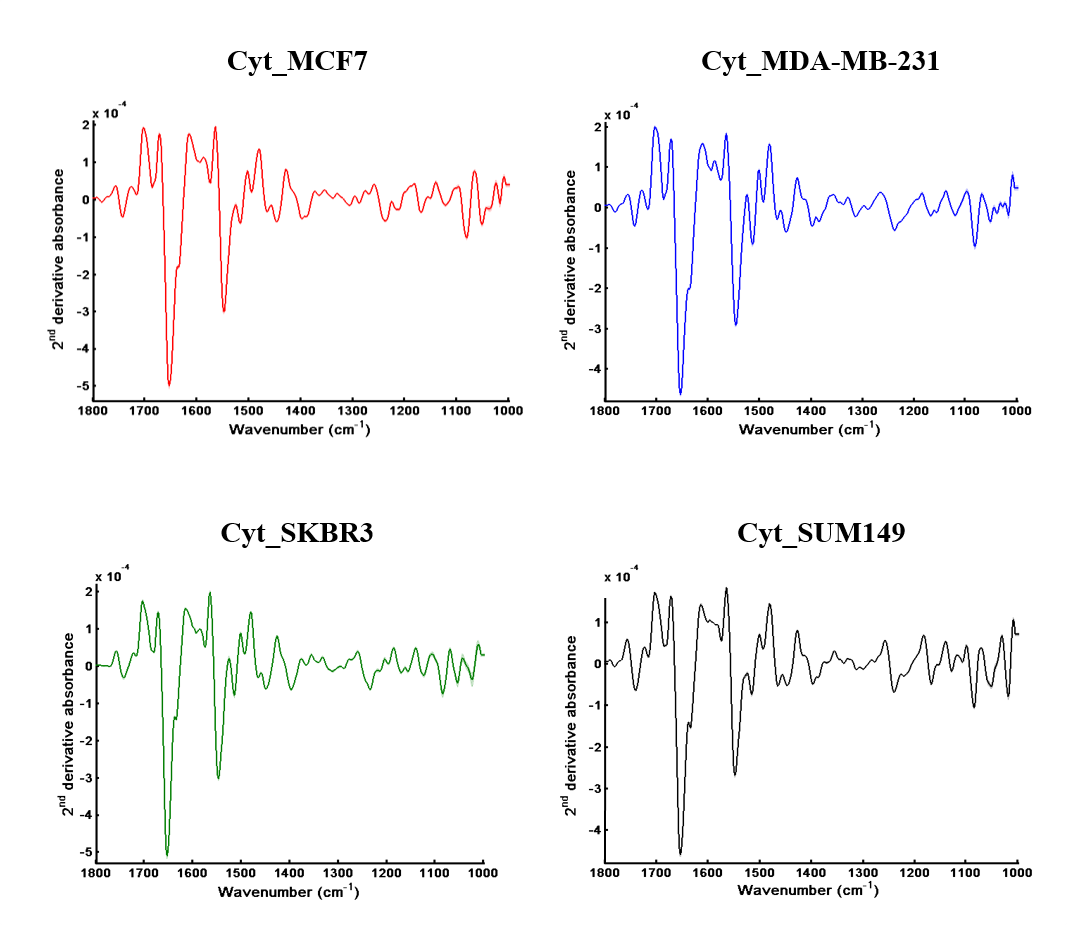

Supplement: Supplementary file 1 [file molecules-25-04300-s001.zip › Figure S6.tif]
